# Maturation is required to model ischemia-reperfusion injury in engineered human cardiac tissues

**DOI:** 10.3389/fbioe.2026.1841042

**Published:** 2026-07-13

**Authors:** Trevor R. Nash, Roberta I. Lock, Vanessa Yi Ran Li, Morgan J. Lamberti, Youngbin Kim, Eloy Sanchez, Sharon Fleischer, Daniel Naveed Tavakol, Gordana Vunjak-Novakovic

**Affiliations:** 1 Department of Biomedical Engineering, Columbia University, New York, NY, United States; 2 Department of Medicine, Columbia University, New York, NY, United States; 3 College of Dental Medicine, Columbia University, New York, NY, United States

**Keywords:** cardiac, iPSC, ischemia reperfusion injury, myocardial infarction, tissue engineering

## Abstract

**Introduction:**

Myocardial infarction remains a leading cause of mortality worldwide, with reperfusion therapy as the clinical standard of care. However, its efficacy is limited by the paradoxical injury caused by ischemia-reperfusion (I/R).

**Methods:**

Micro-sized human engineered cardiac tissues (hECTs) were fabricated using induced pluripotent stem cell (iPSC)-derived cardiomyocytes and primary human cardiac fibroblasts in the previously published milliPillar platform and cultured under four conditions integrating metabolic and electrical maturation strategies: standard B27 medium or metabolic maturation medium (MM), each with or without frequency-ramped electrical stimulation. After 28 days of maturation, tissues were subjected to 6 hours of simulated ischemia followed by reperfusion and were assessed for LDH release, cardiac troponin T (cTnT) release, and contractile function over 7 days post-reperfusion.

**Results:**

Here, we demonstrate that maturation of a human tissue-engineered cardiac model of I/R injury can recapitulate key features of acute ischemic damage, reperfusion injury, and sustained contractile dysfunction. Modulation of engineered tissue phenotype results in distinct differences in susceptibility to injury and pathological fidelity.

**Discussion:**

Notably, electrical stimulation emerges as a critical determinant of I/R injury response, whereas metabolic maturation alone is insufficient to reproduce comparable pathological outcomes. Together, these findings define the structural and functional parameters required to prime cardiac muscle tissues to recapitulate a biologically relevant *in vitro* model of myocardial I/R injury and provide a framework for improving the translational fidelity of engineered cardiac systems.

## Introduction

Acute myocardial infarction (MI) is one of the leading causes of death worldwide, with approximately one million occurring in the United States alone each year (Mechanic et al., 2026; Frank et al., 2012). During MI, loss of blood flow to the infarcted heart results in cardiac ischemia. This acute deprivation of oxygen and nutrients, coupled with accumulation of waste products, triggers cardiomyocyte death and irreversible myocardial damage. The key to limiting myocardial damage and improving prognosis is timely reperfusion and restoration of coronary blood flow (Barron et al., 1998). However, while reperfusion therapy has clear benefits in improving survival rates following MI, its efficacy is limited by the rapid return of oxygen and change in pH to the ischemic environment, which paradoxically exacerbates cardiomyocyte death. This phenomenon, also known as ischemia reperfusion (I/R) injury, may account for up to 50% of the final infarct size and contributes to adverse fibrotic remodeling following MI (Hausenloy et al., 2013). Consequently, understanding the molecular mechanisms underlying I/R injury and developing effective therapeutic strategies to mitigate its impact are of great interest to the clinical community.

In pursuit of this goal, studies conducted in animal models of I/R injury have identified several therapeutic targets, though none have been successfully translated to the clinic (Lara-Pezzi et al., 2015). It is becoming increasingly clear that species-specific variations in cardiac metabolism, electrophysiology, and stress response may be an underlying cause of the failure to translate these potential therapeutics, highlighting the need for models that more accurately mimic the human myocardium. Human induced pluripotent stem cell derived cardiomyocytes (iCMs), whether in monolayer or in spheroid co-cultures, have emerged as promising patient-specific tools to model cardiac pathophysiology *in vitro* (Shi et al., 2017). However, many recently developed *in vitro* models of I/R using iCMs have limited ability to faithfully recapitulate clinically relevant injury phenotypes. These include hallmarks such as acute reperfusion-associated cardiomyocyte injury, sustained reduction in muscle contractile function, and the long-term remodeling of the tissue mediated by the stromal cell populations that results in fibrosis and increased muscle stiffness (Häkli et al., 2021; Yamasaki et al., 2021; Richards et al., 2020). This is likely due to the well-known immaturity of the iCMs used, as immature iCMs are more resistant to ischemic conditions due to their glycolytic metabolism rather than reliance on oxidative phosphorylation (Robertson et al., 2013; Koivumäki et al., 2018; Hidalgo et al., 2018; Van Den Berg et al., 2015).

To overcome these limitations, 3D engineered cardiac tissue models have gained traction for their ability to co-culture multiple cell types along an anisotropically aligned axis and hold capacity for long-term 3D culture for up to several months to enhance iCM cellular maturity (Zhao et al., 2019; Giacomelli et al., 2020). In previous studies conducted by others and ourselves, cardiac tissues were engineered from iCMs and primary human fibroblasts of dermal or cardiac origin, and cultured in RPMI basal medium with B27 supplement (Komae et al., 2017; Mannhardt et al., 2016; Tamargo et al., 2021). iCM phenotypes within engineered cardiac tissue can be substantially matured by manipulation of metabolic substrate composition and electromechanical conditioning (Boudou et al., 2012; Radisic et al., 2004; Feyen et al., 2020; Ronaldson-Bouchard et al., 2018). Recent work by Li et al. demonstrated that integrating both methodologies was effective in promoting further maturation of 2D iCMs specifically in terms of electrophysiology, calcium handling, and alignment (Li et al., 2025). By integrating cellular maturation techniques within 3D microtissues, we explored whether we could not only further improve the biological fidelity of engineered cardiac muscle tissues, but also more accurately recapitulate their responses to I/R injury.

To this end, we investigated both the isolated and combined effects of metabolic modifications to culture medium and electromechanical stimulation on maturation of human engineered cardiac tissue structure and function, and the resulting effects on biologically meaningful recapitulation of I/R injury. We show that coordinated bioenergetic and electromechanical maturation enhanced tissue organization and functional performance compared to tissue culture without stimulation. Furthermore, electrical stimulation was critical to capture key characteristics of I/R injury such as acute myocyte death and reduced functional performance during the days following reperfusion.

## Results

### Experimental design

**FIGURE 1:**
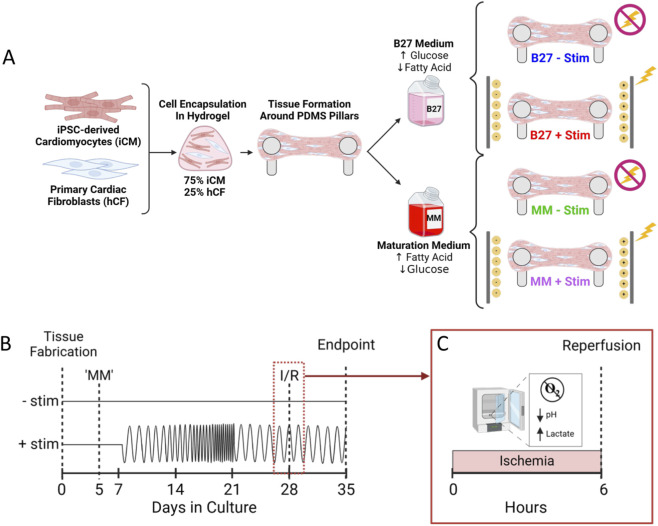
Experimental overview. **(A)** Human iCMs and primary cardiac fibroblasts were encapsulated in fibrin hydrogels around flexible PDMS pillars. After 5 days of initial tissue formation, tissues were either kept in B27 medium or transferred to MM. At day 7, tissues either started a 2-week frequency-ramped electrical stimulation protocol (from 2 Hz to 6 Hz) and then were continuously stimulated at 2 Hz for duration of culture, or kept in culture without electrical stimulation. This resulted in 4 groups of differentially conditioned engineered cardiac tissues (B27-stim, B27+stim, MM+stim, and MM-stim). **(B)** Timeline from tissue fabrication to endpoint, depicting when the switch to MM from B27 occurred (day 5, in the MM+stim and MM-stim groups), the stimulation regimen, and when I/R was conducted during culture (day 28). The timeline of I/R injury is depicted in **(C)**.

Tissues were fabricated in our well established milliPillar platform (Tamargo et al., 2021). Cardiac cells (75% iCM and 25% primary human cardiac fibroblasts, hCF) were encapsulated in fibrin hydrogels and the resulting cell-hydrogel constructs were cultured and stretched between two elastic pillars for 28 days. We investigated different media compositions and electromechanical stimulation conditions to systematically evaluate their effects on tissue maturation and responses to I/R injury (Figures 1A,B). After tissue formation in the standard RPMI medium (days 1–7), the media was switched to either the standard RPMI1640 + B27 (“B27”), traditionally used to culture iCMs in monolayers, or to a lipid-rich and glucose-poor ‘metabolic maturation medium’ (“MM”) that was shown by Feyen and colleagues to effectively initiate a metabolic shift toward oxidative phosphorylation and to better support the high energy demands of maturing cardiomyocytes (Feyen et al., 2020).

We combined the metabolic conditioning with a frequency-ramped electromechanical stimulation regimen, which has been shown to improve tissue structure, contractile function and calcium-handling (“B27+stim”, “MM+stim”), thereby promoting electromechanical maturation essential for recapitulating adult-like cardiac responses. One week after fabrication, stimulation began at 2 Hz and was incrementally ramped by 0.33 Hz per day, to 6 Hz over 2 weeks, after which stimulation returned to 2 Hz for the rest of the culture period, as in our previous studies (Tamargo et al., 2021; Ronaldson-Bouchard et al., 2018). To delineate between the isolated and synergistic effects of the metabolic and electromechanical maturation, we also cultured tissues without electrical pacing (“B27-stim”, “MM-stim”). After 28 days, the tissues were evaluated over the course of I/R (Figures 1B,C).

To simulate I/R, we used a previously developed protocol (Chen and Vunjak-Novakovic, 2019) that recapitulates key pathological features of the ischemic environment, including the low pH (6.4) and elevated lactate and potassium concentrations. We replaced the cell culture medium with a low volume of ischemic medium to promote buildup of metabolic waste products and placed the tissues into a hypoxic chamber (5% CO_2_, 0.1% O_2_) for 6 hours (Supplementary Figures S1A–S1D). Reperfusion was initiated by replacing the culture medium and reoxygenating the environment. Following reperfusion, the tissues were monitored for functional changes and maintained in culture for one more week.

In our *in vitro* studies, functional metrics help compare our engineered tissue data to *in vivo* and clinical metrics (Tamargo et al., 2021; Fleischer et al., 2024). Contractile stress is defined as the peak active tension generated per tissue cross-sectional area during a paced contraction cycle, analogous to left ventricular systolic pressure *in vivo*; following I/R, contractile force is markedly reduced due to myofilament calcium desensitization and sarcomeric protein degradation. Contraction velocity as measured here is the velocity of the pillar heads in um/s, which reflects the rate of force development (dF/dt) during systole since there is a linear relationship between force and displacement. This parameter is impaired post-I/R due to reduced ATP availability and oxidative modification of contractile proteins. In addition, relaxation velocity reflects the rate of force decline during diastole, which depends on SERCA2a-mediated calcium reuptake into the sarcoplasmic reticulum; impaired relaxation velocity is a hallmark of reperfusion-induced diastolic dysfunction *in vivo*.

### Modulation of engineered cardiac tissue structure and function

**FIGURE 2:**
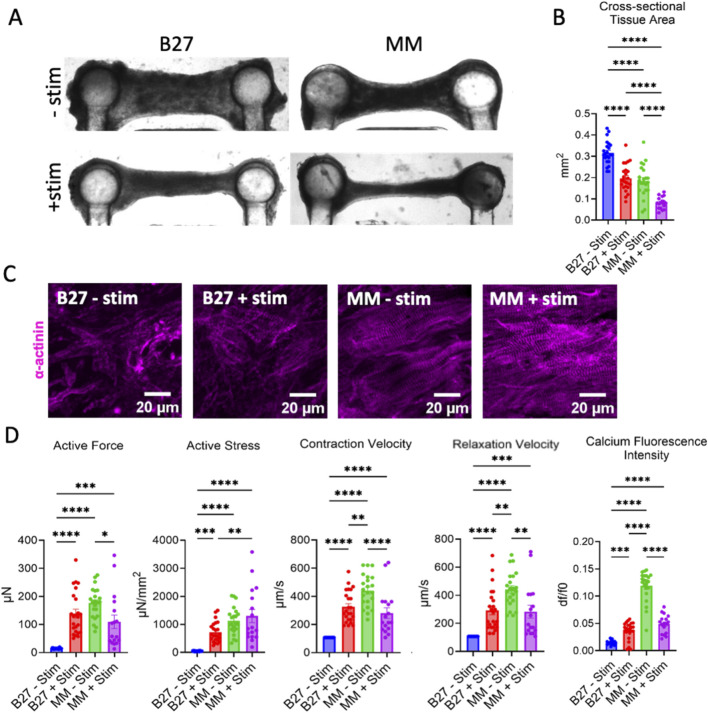
External stimuli alter engineered tissue structure and function. **(A)** Brightfield images of tissues cultured under the 4 different culture conditions. **(B)** Quantitative assessment of tissue cross-sectional area. **(C)** Whole mount immunofluorescence images of engineered tissues alpha-actinin. **(D)** Quantitative assessment of active force, active stress, contraction velocity, relaxation velocity, and calcium intensity (n = 15–25 individual tissues, across multiple independent experiments). All data is shown as mean ± SEM. *p < 0.05, **p < 0.01, ***p < 0.001, ****p < 0.0001 and statistical analysis was performed using ordinary one-way ANOVA corrected for multiple comparisons using Tukey’s hypothesis testing.

Prior to exposing the tissues to simulated I/R, we compared the tissues engineered under different culture conditions. At the 28-day mark, we observed pronounced effects on tissue structure (Figure 2). For both B27 and MM groups, the electromechanically stimulated (+stim) tissues exhibited decreased cross-sectional area compared to -stim tissues. In both the +stim and -stim groups, tissues cultured in MM exhibited the greatest decrease in cross-sectional area compared to their B27 counterparts (Figures 2A,B). To investigate the structural properties underlying these differences, tissues were stained for α-actinin to evaluate cardiomyocyte sarcomere organization (Figure 2C). Notably, qualitative assessment of the MM groups showed significantly increased organization and alignment of α-actinin compared to those cultured in B27, regardless of stimulation.

The structural differences were associated with the differences in contractile tissue function. Measurements of the active contractile properties (e.g., active force, active stress, work, and contraction and relaxation dynamics) revealed several interesting trends. Tissues cultured in B27 medium showed markedly increased force generation in the B27+stim group compared to the B27-stim group (Figure 2D). Additionally, both the contraction and relaxation velocity were also increased in the B27+stim group compared to B27–stim group. Overall, these results indicate that for tissues cultured in B27 medium, the ramped electrical stimulation during 28 days of culture increased the functional abilities of the tissue, consistent with the previous reports (Tamargo et al., 2021; Ronaldson-Bouchard et al., 2018).

Interestingly, the effects of the combined metabolic and electrophysiological stimulation were different for the MM groups. Overall, MM-stim outperformed MM+stim, exhibiting significantly higher active force production and active stress, higher contraction, and relaxation velocity (Figure 2D). When compared to the B27+stim groups, the MM+stim tissues displayed a similar functional performance regardless of culture media. However, all groups performed better than the B27-stim group, which was representative of traditional baseline culture conditions for iCM. Together, these results demonstrate that the application of different combinations of extrinsic stimuli to an engineered cardiac tissue leads to distinct differences in tissue structure and function.

### I/R induced acute cardiac injury in tissues matured with electromechanical stimulation

Following optimization of the I/R setup, tissues from all culture groups (B27-stim, B27+stim, MM-stim, MM+stim) were subjected to 6 h of simulated ischemia, as described previously (Chen and Vunjak-Novakovic, 2019), followed by reperfusion, to assess how each of these four different culture conditions influenced the recapitulation of acute cardiac injury.

Cellular damage due to ischemic injury was evaluated by assessing LDH release immediately following simulated ischemia (or 6 h of culture under standard conditions for normoxic controls), prior to reperfusion. Analysis revealed that +stim groups had significant increases in LDH release in response to ischemia when compared to their normoxic controls (Supplementary Figure S2A). In comparison, the MM-stim group did not have a significant increase in LDH release following simulated ischemia. Direct comparison of the magnitude of LDH release in response to ischemia across the different maturation groups revealed that the MM+stim group had the most robust response, that was not significantly different from that of the B27+stim group (Supplementary Figure S2B). The subsequent acute reperfusion injury was assessed by measuring LDH release 1 h following reperfusion. All four groups exhibited trends of increased LDH release compared to the corresponding normoxic controls, with statistically significant differences observed only for the +stim groups, similar to the trends seen for the ischemic injury (Supplementary Figure S2C). Interestingly, the magnitude of cell damage at 1 h following reperfusion revealed no significant differences between the groups (Supplementary Figure S2D). As expected, cell and tissue damage were observed both during the ischemic period and the reperfusion, in line with the previous studies of I/R injury (Chen and Vunjak-Novakovic, 2019).

**FIGURE 3:**
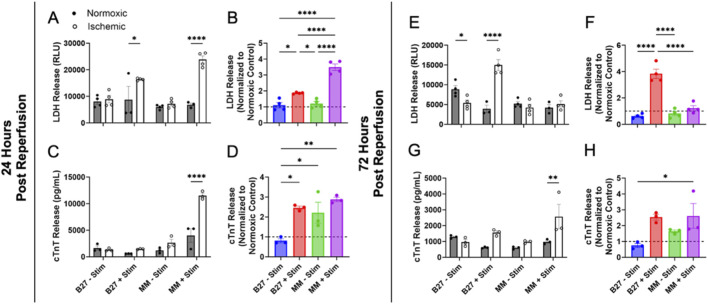
Tissue pre-conditioning impacts extent of cellular damage from reperfusion injury. Cellular damage 24 h following reperfusion is quantified by LDH **(A,B)** and cardiac troponin (cTnT) **(C,D)** release in tissues subjected to ischemia compared to their normoxic controls for all conditioning groups, or normalized to their respective controls **(B,D)**. Cellular damage 72 h following reperfusion is similarly quantified by LDH release in **(E,F)**, and cTnT release in **(G,H)**, respectively. Data shown is from n = 3-6 tissue replicates and mean ± SEM. *p < 0.05, **p < 0.01, ***p < 0.001, ****p < 0.0001 and statistical analysis was performed using ordinary one-way ANOVA corrected for multiple comparisons using Tukey’s hypothesis testing.

Additional supernatant samples were then collected 24 h and 72 h after reperfusion to assess whether the damage from reperfusion injury continued over an extended period of time. At 24 h only, B27+stim and MM + stim groups showed significant increases in LDH released from reperfused ischemic tissues relative to their normoxic controls, though the magnitude of the relative LDH release from the MM+stim group exceeds even that of B27+stim (Figures 3A,B). Cardiac troponin T (cTnT) release, a marker of cardiomyocyte death, was measured 24 h after reperfusion and showed similar trends, with significant differences observed only in the MM+stim tissues (Figure 3C). Evaluation of the relative cTnT levels showed increased release across all groups compared to the B27-stim group, with the greatest magnitude again observed in the MM+stim tissues. This pattern was consistent with LDH release at 24 h.

At 72 h, measurements of LDH and cTnT showed no further increase in cell damage, with only minimal differences between normoxic and ischemic groups (Figures 3E–H). Only the B27+stim group showed significantly higher LDH release from the ischemic group after 72 h (Figures 3E,F); interestingly, only MM+stim demonstrated increased cTnT release from the ischemic group compared to the normoxic controls, although the values for both cases were decreased compared to those observed at the 24 h timepoint (Figures 3G,H). Both timepoints post-reperfusion demonstrated similar trends, given that the +stim groups exhibited the greatest magnitude of injury. Furthermore, comparison of the 1-h, 24-h, and 72-h post-reperfusion timepoints indicates that the acute onset of cellular damage within the tissues occurs within the first 72 h, which correlates with clinical timeframes (Hausenloy et al., 2013).

### Reduced contractile function following I/R in electrically stimulated tissues

Assessment of the effect of simulated I/R on contractile function of tissues matured under different conditions was also conducted via non-invasive, real-time imaging, established in our previous studies (Tamargo et al., 2021). All tissues were imaged prior to ischemia and serially over time after injury for up to 1 week following reperfusion. Functional changes due to I/R damage were evaluated by measuring active force, passive tension, and contraction and relaxation velocities.

**FIGURE 4:**
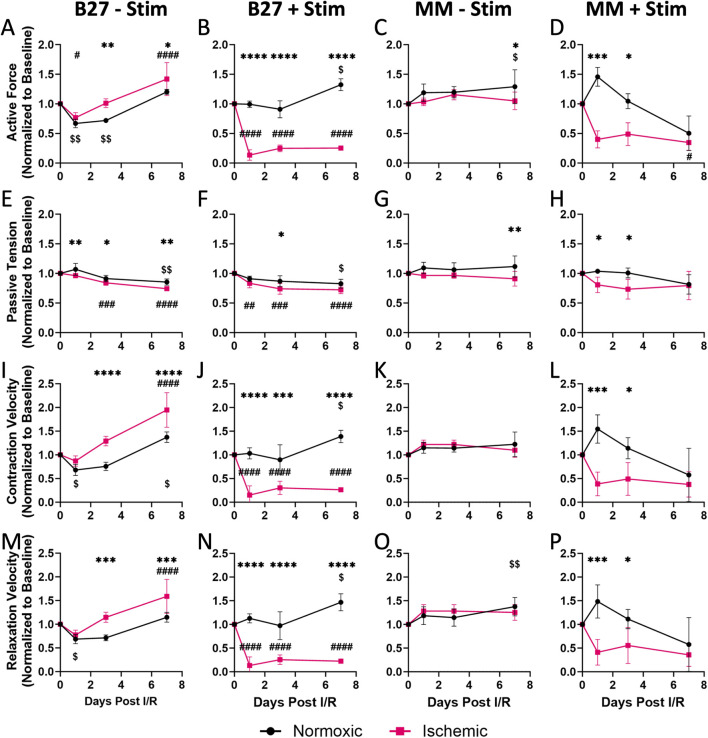
I/R results in sustained contractile dysfunction. Changes in contractile function due to I/R injury across 7 days following injury for B27-stim, B27+stim, MM-stim, and MM+stim tissue groups respectively by quantifying active force (**A–D**), passive tension (**E–H**), contraction velocity (**I–L**), and relaxation velocity (**M–P**). Data shown from n = 3-4 tissue replicates, normalized to baselines, and analyzed using two-way ANOVA, corrected with Sidak multiple comparisons. Data is shown as mean ± SEM. * denotes significant difference between ischemic and normoxic groups at a specified time point. # denotes significant difference within the ischemic group between a specified time point and baseline. $ denotes significant difference within the normoxia group between a specified time point and baseline. *,#,$ p < 0.05, **,##,$$ p < 0.01, ***,###,$$$ p < 0.001, ****,####,$$$$ p < 0.0001. Baselines were defined as absolute values of each parameter after tissue maturation but prior to ischemic injury, individually defined for each tissue.

Following I/R, a significant reduction in the active force was observed in tissues conditioned with electromechanical stimulation (B27+stim and MM+stim) (Figures 4A–D; Supplementary Figures S3A,B), and the effect was sustained. In tissues where this functional impairment occurred, the deficit was sustained over 72 h of culture post I/R, without evidence of recovery. Interestingly, passive tension was comparable for normoxic and ischemic conditions in all groups (Figures 4E–H), and timepoints (Supplementary Figures S3C,D). As expected, the contraction and relaxation velocities showed similar trends to the active force production following simulated I/R, with +stim groups exhibiting reduced contraction and relaxation speeds following injury that correlate with the diminished active force capacity observed (Figures 4I–P; Supplementary Figure S3E–H).

## Discussion

In this study, we evaluated complementary strategies for maturing iPSC-derived engineered human cardiac tissues (frequency-ramped electrical stimulation and/or metabolic substrate modulation via lipid-rich, glucose-poor maturation medium) and examined how these conditions influenced the tissues’ capacity to recapitulate physiological features of I/R injury. The rationale for using multiple maturation strategies stems from the well-known limitation that most *in vitro* models using iPSC-derived cardiomyocytes fail to faithfully model adult cardiac injury due to their immaturity (Koivumäki et al., 2018). Two-dimensional iPSC-CM monolayers have well-documented limitations in modeling I/R injury due to their immature metabolic phenotype and lack of three-dimensional tissue architecture. Three-dimensional organoid and cardiac microtissue models provide improved structural organization but often lack the electromechanical conditioning and real-time force measurement capabilities central to the present work. The milliPillar platform uniquely integrates frequency-ramped electrical stimulation and non-invasive contractile force monitoring in a three-dimensional architecture. Nonetheless, this advantage should be understood in comparative rather than absolute terms, acknowledging that direct head-to-head benchmarking remains an important direction for future validation (Häkli et al., 2021; Hidalgo et al., 2018; Häkli et al., 2022; Gruszczyk et al., 2022; Correia et al., 2018; Daily et al., 2015; Zhang et al., 2013). In this work, we demonstrate that electrical stimulation produces a functionally distinct conditioning state that primes tissues for injury, rather than simply augmenting contractile output at baseline.

We focused on two different methods known to advance the phenotype of iPSC-derived cardiomyocytes, electromechanical and metabolic maturation, to better understand how they may differentially affect engineered cardiac muscle structure and function in homeostasis, injury, and recovery. Applying a frequency-ramped electrical stimulation regimen to engineered tissues has been shown to improve the sarcomeric organization and force production (Zhao et al., 2019; Ronaldson-Bouchard et al., 2018). However, this regimen has previously only been applied to tissues cultured in the traditional RPMI supplemented with B27 that is commonly used for the culture of iCMs. More recently, Feyen et al. demonstrated that iCMs could be metabolically matured by supplementation with oxidative substrates, such as fatty acids, to shift cardiomyocytes toward a more adult-like metabolic phenotype (Feyen et al., 2020). Consistent with these reports, we observed that electrical stimulation improved force production in B27-cultured tissues, and that metabolic maturation enhanced sarcomeric alignment and contractile performance relative to unstimulated controls (Feyen et al., 2020; Li et al., 2025).

Unexpectedly, the simultaneous application of both maturation strategies did not yield synergistic improvements. Although tissues cultured in maturation medium and subjected to stimulation exhibited sarcomere alignment comparable to metabolic maturation alone, the force production was reduced relative to MM-stim tissues and functionally similar to B27+stim tissues. This finding suggests that concurrent electromechanical and metabolic conditioning may impose competing or excessive energetic demands that limit functional gains. One possibility is that both interventions independently increase metabolic load, and that their combination surpasses the energetic capacity of the tissue under steady-state culture conditions. More broadly, electrically stimulated cardiomyocytes operate with increased energetic demand due to elevated calcium cycling and actomyosin ATPase activity required to sustain paced contractions. The simultaneous shift to oxidative fatty acid metabolism imposed by MM, while beneficial under basal conditions, may impose an increased energetic burden when combined with the elevated mechanical workload of electrically stimulated tissues, potentially limiting net functional gain. This is consistent with literature demonstrating that the energetic cost of contraction in mature, oxidative cardiomyocytes is substantially higher than in glycolytic fetal-like cells, and that forced reliance on fatty acid oxidation can impair function under high-demand conditions (Feyen et al., 2020). We acknowledge that this interpretation remains mechanistic speculation in the absence of direct energetic measurements and have framed this as a hypothesis warranting future investigation. Future studies incorporating metabolic flux‐based energetic profiling under paced conditions, combined with calcium imaging, would be appropriate to directly test this mechanism. Sequential implementation such as metabolic priming (i.e., a 1:1 B27 and MM intermediate) followed by electromechanical conditioning may also better support progressive adaptation and warrants future investigation.

Furthermore, contractile force represents only one dimension of cardiomyocyte maturation. The absence of functional synergy does not preclude potential additive effects on other maturation axes, including transcriptional remodeling, metabolic flux, electrophysiological stability, or calcium handling, which were not comprehensively evaluated in this study. Both metabolic substrate modulation and ramped pacing have been independently reported to enhance oxidative phosphorylation and mitochondrial maturation (Feyen et al., 2020; Ronaldson-Bouchard et al., 2018; Li et al., 2025). However, we did not directly assess metabolic state in tissues exposed to combined conditioning. Comprehensive metabolic profiling such as assessment of mitochondrial respiration, substrate utilization, and redox balance may be critical to elucidating the mechanistic basis for the differential responses to simulated I/R observed in this study.

In characterizing the tissue injury induced by I/R, we employed 0.1% O_2_ rather than the conventionally used 1% O_2_ because cytochrome c oxidase has an extremely low K_m_ for O_2_ and can sustain mitochondrial oxidative phosphorylation at oxygen tensions well below 1%, meaning cardiomyocytes at 1% O_2_ are not in a true ischemic metabolic state. This distinction is particularly critical for metabolically mature cardiomyocytes, which rely heavily on oxidative phosphorylation. By contrast, 0.1% O_2_ approaches anoxia and more faithfully recapitulates the oxygen environment of occluded myocardium *in vivo* (Gruszczyk et al., 2022). Electrical stimulation was maintained throughout the reperfusion phase to mirror *in vivo* physiology. In the *in vivo* setting, continuous cardiac contraction and the associated calcium cycling and metabolic demands are maintained during and after an ischemic event. Discontinuing pacing during reperfusion would therefore introduce an artificial condition that diverges from clinical reality. Our experimental design specifically models the electromechanical state of the reperfused heart, and continuous pacing is integral to that objective. We found that after 6 h of ischemia, the +stim tissue groups not only sustained more significant ischemic injury, but also greater acute and sustained reperfusion injury compared to the -stim groups. Analysis of tissue function was consistent with reports of acute cell damage, and revealed impairment of contractile function in +stim tissue groups for up to 72 h. Importantly, this prolonged functional impairment was similar to the clinical course of some patients who develop chronic systolic dysfunction (i.e., heart failure with reduced ejection fraction, HFrEF) following myocardial infarction and subsequent reperfusion.

Within the +stim groups, metabolic conditioning with either B27 or MM did not significantly alter injury severity. Although MM-stim tissues demonstrated improved baseline sarcomere alignment and some enhancement of contractile function prior to injury, these gains did not translate into increased susceptibility to ischemia-reperfusion stress. This was unexpected, as we presumed that MM-cultured tissues, which rely on oxidative phosphorylation, would exhibit more severe injury. Instead, the application of electrical stimulation emerged as the dominant factor that increases tissue vulnerability. A potential explanation is that re-initiation of 2 Hz pacing during reperfusion imposed additional energetic and contractile demand, limiting recovery from acute stress. Collectively, these findings suggest that electromechanical maturation, rather than metabolic modulation, is a dominant determinant of I/R susceptibility in this model. More broadly, our results suggest that not all forms of cardiomyocyte maturation are important for disease modeling, and that the improvements in baseline structure or function do not necessarily enhance pathological fidelity. The mechanisms underlying the heightened susceptibility of electrically conditioned tissues remain to be defined. The collected data suggest the possible roles of altered excitation-contraction coupling, calcium handling, and increased energetic demand.

We specifically explored two unique maturation protocols for enhancing the functionality of engineered cardiac tissues and the physiological relevance of their responses to injury. Moving forward, the inclusion of tissue-resident cardiac macrophages or endothelial cells may be the next step in advancing the biological fidelity of engineered tissue models of I/R, as these supporting cells in the cardiac muscle are known sensors of tissue stress (Koivumäki et al., 2018). *In vivo* I/R injury triggers innate immune responses beginning within minutes of reperfusion, beginning with CCR2-resident cardiac macrophages rapidly shifting toward pro-inflammatory activation, releasing TNF-α, IL-1β, and IL-6 cytokines, and amplifying cardiomyocyte death through paracrine signaling and exacerbating calcium overload (Landau et al., 2024; Lock et al., 2024). Subsequent recruitment of CCR2+ monocytes and neutrophil infiltrate the injured tissue and contribute to remodeling of the tissue in the days to weeks post-injury. The absence of these myeloid populations in our model means that the injury phenotype we observe reflects the intrinsic cardiomyocyte and fibroblast responses to ischemic stress, but does not capture the full inflammatory amplification that occurs *in vivo*.

Several important limitations of this study should be noted. Assessment of I/R injury was primarily restricted to LDH release, cTnT release, and contractile parameters. These represent clinically validated biomarkers and direct functional correlates of cardiac performance. However, additional endpoints including markers of apoptosis (e.g., cleaved caspase-3), necroptosis assessment, extracellular matrix (ECM) remodeling, and extended longitudinal follow-up beyond 7 days would further enrich the dataset. In addition, targeted molecular characterization of mitochondrial health and transcriptomic signatures at each stage post-I/R injury would provide a deeper understanding of more immediate phenotypic changes to the cardiac muscle. These are planned as future directions, particularly for studies modeling post-infarction remodeling and fibrosis, which are distinct long-term pathological processes resulting from the acute I/R injury phase that is the focus of the current study. Additionally, all experiments were performed with a single iPSC line. iPSC line-to-line variability is a known source of biological heterogeneity, and the generalizability of our findings across additional donor lines remains to be established. We note that the main conclusions, particularly the dependence of I/R susceptibility on electrical maturation state, are supported by replicate, independent experiments, but multi-donor validation is an important direction for future investigation.

In conclusion, we report that a human tissue-engineered model of myocardial I/R injury can capture some of the key physiological features of acute myocyte injury as well as prolonged contractile dysfunction. By applying multiple maturation techniques, individually and in combination, we were able to show that the specific conditioning strategies can differentially impact tissue structure, baseline function before injury, and their responses to injury. Specifically, we found that tissue maturation by electrical stimulation is a critical component for recapitulating clinically relevant susceptibility to I/R, and that metabolic maturation alone does not confer the same pathological fidelity. Moving forward, further refinement of tissue-engineered models of I/R will benefit from incorporating supporting cell types and optimizing maturation protocols. These advances will enhance our ability to uncover mechanisms of injury and guide the development of novel therapeutic strategies.

## Materials and methods

### Cell sourcing

hiPSCs were sourced from the previously generated WTC11-GCaMP6f hiPSC line (and an unlabeled version of the line, WTC11), both gifted from Dr. Bruce Conklin from the Gladstone Institute (Huebsch et al., 2016). The WTC11-GCaMP6f line contains a constitutively expressed GCaMP6f calcium-responsive fluorescent protein inserted into a single allele of the AAVS1 safe harbor locus, enabling real-time and label-free visualization of calcium handling (Daily et al., 2015; Chen et al., 2013).

### iPSC cell culture

All hiPSCs were cultured in mTeSR Plus medium (STEMCELL Technologies, 100–0276) on tissue culture plates coated with Matrigel (Corning, 354230; 1:100) and passaged every three to 4 days with 0.5 mM EDTA (Thermo Fisher Scientific, 15575) in PBS (Corning, 21–040). For the first 24 h after passaging, 5 μM Y-27632 dihydrochloride (Tocris Bioscience, 1254), was added to the culture medium. hiPSCs were karyotyped and regularly tested for *mycoplasma* contamination.

### iCM differentiation

Cardiomyocytes were differentiated from hiPSCs by adapting the protocol developed by Burridge et al. (2014). iPSCs were replated at a density of 250,000 cells/cm^2^ on tissue culture plates coated with Matrigel in mTeSR Plus medium with 5 μM Y-27632 dihydrochloride. 24 h after plating, mTeSR Plus media was refreshed. Following that, for day 0 of the differentiation, cells were switched to CDM3 media (RPMI1640 (Gibco, A41923-01), 0.5 mg/mL human Albumin (Sigma, A9731-1G), and 213 ug/mL L-ascorbic acid 2-phosphate (Sigma, A8960)), with 5 uM CHIR 99021 (Tocris, 4423). On day 1, medium was changed to CDM3. On day 2, medium was changed to CDM3 supplemented with 2uM Wnt-C59 (Tocris, 5148). On day 4, medium was changed to CDM3, and refreshed every other day until day 10, when medium was switched to B27 media (RPMI 1640 + B27 supplement (Thermo Fisher Scientific, 17504044). Contracting cells were noted as early as day 7, but typically around day 10.

### Cardiac fibroblast culture

Primary human ventricular cardiac fibroblasts (NHCF-V, Lonza, CC2904) were cultured according to the manufacturer’s protocol with Fibroblast Growth Medium 3 (PromoCell, C-23130). Fibroblasts were used for engineering tissues between passage 3 and passage 5. Cells were generated from healthy donors with no history of cardiovascular disease.

### Maturation media formulation

The metabolic maturation medium (MM) used in this study was adopted from Feyen et al. (2020). The MM consists of glucose-free DMEM (containing Ca^2+^ 1.80 mM, K^+^ 5.33 mM, Na^+^ 110.34 mM; with standard amino acids including Glycine, L-Arginine, L-Cystine, L-Glutamine, L-Histidine, L-Isoleucine, L-Leucine, L-Lysine, L-Methionine, L-Phenylalanine, L-Serine, L-Threonine, L-Trypt\ophan, L-Tyrosine, and L-Valine; and vitamins including Choline, D-Calcium pantothenate, Folic Acid, Niacinamide, Pyridoxine, Riboflavin, Thiamine, and i-Inositol), supplemented with B27 (which contributes extra amino acids: L-Asparagine, L-Aspartic acid, L-Glutamic Acid, L-Proline; and extra vitamins: Biotin, Vitamin B12), adult cardiomyocyte media supplements (L-carnitine, taurine, creatine, ascorbic acid), metabolic substrates (Glucose 3 mM, L-lactate 10 mM, AlbuMax lipid-rich BSA), and Knockout serum replacement (1%). This formulation was shown by Feyen et al. to significantly enhance basal and maximal oxygen consumption rate (OCR) measured by Seahorse extracellular flux analysis, increase glycolytic capacity (ECAR), elevate mitochondrial content (quantified by Tom20 flow cytometry and immunostaining), improve Ca^2+^ cycling via functional SERCA2a/PLN assays, upregulate fatty acid metabolism genes by RNA sequencing, and improve contractile force, sarcomere structure, and long-term survival in engineered heart tissues. As the metabolic characterization of this medium has been thoroughly established in that prior work, we did not replicate Seahorse measurements within the milliPillar system in the current study; however, we acknowledge this as an important future direction.

### Generation of human engineered cardiac tissues (hECT)

hECTs were formed as previously described (Tamargo et al., 2021). Briefly, the culture platforms were made by casting PDMS in custom-milled molds containing carbon rod electrodes. The resulting platforms were plasma-bonded to glass slides and the electrodes were connected to platinum wires to facilitate electrical stimulation during culture. The complete platform was autoclaved prior to use.

To fabricate the tissues, iPSC-derived cardiomyocytes and human primary cardiac fibroblasts were dissociated using 10X TrypLE Select Enzyme (Fisher, A1217702) for 5 min at 37 C. Cells were counted and resuspended at a concentration of 550,000 cells total per tissue in a solution of 3 mg/mL fibrinogen in B27 medium, with iCM composing 75% of total cell number, and hCF composing the remaining 25% (a ratio empirically optimized in prior work from our laboratory using the milliPillar platform) (Tamargo et al., 2021; Fleischer et al., 2024). For each tissue, 12 μL cell suspension was mixed with 3 µL thrombin (2.5 U/mL) in a well of the bioreactor to form the tissue around the pillars. The tissues were placed in a 37 °C incubator for 30 min for the hydrogel to crosslink, then B27 media supplemented with 10uM Y-27632 dihydrochloride and 5 mg/mL 6-aminocaproic acid (Sigma-Aldrich, A7824) was added to each well containing a tissue. 1–3 h post fabrication, tissues were manually detached from the sides of the well and the underlying glass slide using a 26-gauge needle.

On day 1 following fabrication, Y-27632 dihydrochloride was removed from the medium, and tissues were manually detached a second time. Medium was changed every day until day 5, when the 6-aminocaproic acid was removed from the medium. On day 5, tissues that were to be culture in B27 remained in B27, but tissues that were to be culture in MM were changed into a combination medium of 50% B27% and 50% MM media to facilitate transition to a new media composition. On day 6, tissues to be cultured in MM changed from the combination medium to a complete MM culture medium composition. From day 7 until the end of culture, the appropriate cell culture medium was refreshed every other day. Additionally, on day 7, electrical stimulation of the ‘+stim’ tissues was started using a custom Arduino-based electrical stimulator as previously described (Tamargo et al., 2021; Ronaldson-Bouchard et al., 2018), which stimulated tissues using a 5 V/cm biphasic pulse (2 ms pulse length) at 2 Hz frequency. Tissues were subjected to a 2-week ramped electrical stimulation regimen, where pacing began at a 2 Hz frequency that increased every 24 h by 1/3 Hz until reaching 6 Hz. After reaching 6 Hz, frequency was reduced back to 2 Hz, which was maintained until the endpoint.

### Contractile function analysis

Assessment of tissue contractile function and force generation capacity was conducted by capturing video of tissue contraction while stimulated at various voltages and frequencies. Tissues were placed in a live-cell chamber (SFX Temp and CO_2_ Stage Top Incubator, Tokai Hit) for controlled environment set to 37 °C and 5% CO_2_, and imaged using a sCMOS camera (Zyla 4.2, Andor Technology) connected to an inverted microscope (IX-81, Olympus). Brightfield videos were acquired at 20 frames per second for 2400 frames using an Arduino-based electrical stimulator and custom program to stimulate the cardiac tissues from 0 Hz - 4 Hz. Force generation was determined by analyzing pillar deflection using a custom Python script as previously described (Tamargo et al., 2021). Briefly, the script identified the location of the pillar heads to determine deflection of the pillar during tissue contraction, and force was calculated by multiplying the displacement by a coefficient determined by the force displacement calibration curve that was empirically determined for the platform’s pillars via mechanical testing. Additionally, analysis of the deflection trace yields many other metrics of contractility, such as total force, passive force, active force, total stress, passive stress, active stress, contraction velocity, and relaxation velocity. Tissues that did not capture at 1 Hz stimulation at the end of the ramped stimulation period were excluded from further analysis. Our analysis pipeline is now available as an open source platform known as BeatProfiler, a suite of tools to analyze cardiac muscle tissue function *in vitro* (Kim et al., 2024).

### Calcium signaling analysis

The use of GCaMP6f fluorescent protein in the iCMs enabled visualization of calcium transients without need for addition of dyes. Tissues were imaged with the same set up as described for force generation analysis, with fluorescence visualized using a standard GFP filter set. Tissues were electrically stimulated at 1 Hz and videos were acquired at 20 frames per second to measure calcium flux. Calcium signals were analyzed as previously described (Tamargo et al., 2021). Briefly, the calcium transient within the tissue was assessed by tracking average pixel intensity over time, creating a trace which was then corrected to account for the gradual decay of the baseline fluorescence that occurs due to photobleaching effect using a custom Python script.

### Whole mount immunofluorescence imaging

To stain engineered cardiac tissues, the PDMS pillars were gently disconnected from the rest of the bioreactor and the intact tissues were moved to a well-plate for fixation and staining. Tissues were fixed with 4% Paraformaldehyde Solution in PBS (Santa Cruz Biotechnology, sc-281692) for 20 min at room temperature, permeabilized in 0.25% Triton X-100 (Sigma, X100-100 mL) for 20 min at room temperature, and then blocked with 5% BSA (Sigma, A7906-500G) in PBS for 2 h at room temperature. For staining with primary antibodies, tissues were incubated with α-actinin (1:100, Sigma, cat. No. A7811), Vimentin (1:1000, Abcam, ab24525), and myosin light chain 2 (MLC2v) (1:400, Abcam, ab79935) in 1% BSA in PBS overnight at 4 C, and residual stain was then washed-out using PBS. Tissues were then incubated with the appropriate secondary antibody in 1% BSA in PBS for 1 h at room temperature, followed by washing in PBS. Nuclei were stained using DAPI (1:1000, BD Biosciences, 564907) in PBS for 15 min at room temperature, followed by PBS washing. Finally, the pillars were gently removed from the tissues using forceps and the tissues were then embedded in ProLong Diamond Antifade Mountant (Invitrogen, P36931) in CoverWell incubation chambers (Grace Bio-labs, 645501) sealed with a glass coverslip. Whole mount z-stack images were acquired using either a Nikon Ti Eclipse inverted microscope for spinning-disk confocal microscopy or a Nikon Ti2 inverted microscope with AXR resonant spectral scanning confocal unit. Images were then processed in ImageJ to create z-stack maximum projection images.

### Simulated ischemia and reperfusion

Ischemia and reperfusion were simulated as previously described (Chen and Vunjak-Novakovic, 2019). Briefly, engineered tissues were rinsed with PBS and then 100 uL of ischemic medium (described below) was added to each culture well in the milliPillar bioreactor. The bioreactors were then placed in a humidified hypoxic incubator (New Brunswick Galaxy 48 R) set to 0.1% O_2_, 5% CO_2_, and 37 °C for 6 hours to simulate ischemia. Nitrogen gas was used to purge the incubator of O_2_. Normoxic control tissues were maintained in 400 uL regular culture medium (e.g., maturation medium or RPMI-B27) in the standard 5%CO_2_, 37 °C incubator. After 6 hours, the bioreactors were removed from the hypoxic incubator, ischemic media was removed from each well and collected, and 400 uL of the appropriate regular culture medium was added to each well to simulate reperfusion. Culture media was also changed in the control reactors at the same time.

### Ischemic media formulation

To create ischemic medium solution, the components listed in Supplementary Table S1 were combined at the appropriate concentrations in distilled UltraPure water, and the pH was then adjusted by adding 1.0 M HCl dropwise until pH = 6.4. Completed ischemic media formulation was then sterile filtered and stored at 4 °C until use. Media pH was confirmed prior to each experiment.

### Environmental sensing

Optical pH (PreSens Precision Sensing GmbH, SP-HP5-F100-D5-US) and dissolved O_2_ (PreSens, SP-PSt3-NAU-D5-YOP) sensors were incorporated into empty milliPillar bioreactor wells by using a 5 mm biopsy punch to create a gap in the PDMS at the bottom of each well into which the sensor (5 mm in diameter) could be placed securely without the need for adhesives. A fiber optic cable (PreSens, POF-L2.5-1SMA) was secured below each optode and connected to the appropriate control box (O_2_ sensor: Fibox 4, PreSens; pH sensor: pH-1 Mini v2, PreSens), according to the manufacturer’s instructions. The control boxes were connected to a PC running Windows 10. PreSens Measurement Studio version 2 software was used to acquire and store measurements. Sensors were calibrated before use according to the manufacturer’s protocol.

To monitor O_2_ and pH during the ischemic period, the optodes in the bioreactor were covered with the type and volume of media corresponding to the experimental wells with tissues, then placed within the hypoxia chamber. Dissolved oxygen concentration and pH were continuously monitored in real-time and recorded. Ambient temperature within the chamber was also recorded for compensation of the optode signals.

### cTnT ELISA

To quantify human cardiac troponin T release from tissues, supernatant was collected at different timepoints following a 24-h period of incubation with the tissues and frozen and stored at −20 C for later use. Upon thaw, supernatant samples were used undiluted in the human cardiac troponin T ELISA kit (Abcam, ab223860), and assay was performed according to manufacturer’s instructions.

### LDH release assay

To quantify LDH release from tissues, supernatant was collected from tissues following a 24-h period of incubation and diluted 1:20 into LDH storage buffer (200 mM Tris-HCl (pH 7.3), 10% Glycerol, 1% BSA) as directed by manufacturer’s protocol, then subsequently frozen and stored at −20 C for later use. Upon thaw, diluted supernatant was used to quantify the amount of LDH present in the tissue supernatant using LDH-Glo Cytotoxicity Assay (Promega, J2380), carried out following manufacturer’s instructions.

### Statistical analysis

All analyses were conducted in GraphPad Prism version 10. Unless otherwise indicated, comparisons between groups with only a single independent variable were made using an ordinary one-way ANOVA corrected for multiple comparisons using Tukey’s hypothesis testing, and comparisons between groups with two independent variables were made by conducting a two-way ANOVA corrected for multiple comparisons using Sidak’s method. Unless otherwise indicated, all data is shown as mean ± SEM. *p < 0.05, **p < 0.01, ***p < 0.001, ****p < 0.0001.

## Data Availability

The original contributions presented in the study are included in the article/Supplementary Material, further inquiries can be directed to the corresponding author.
