# Supplementary material for: Maturation is required to model ischemia-reperfusion injury in engineered human cardiac tissues
Source: Front Bioeng Biotechnol. 2026 Jul 13;14:1841042. doi: 10.3389/fbioe.2026.1841042 (PMC13402417; doi:10.3389/fbioe.2026.1841042)
Supplement: Supplementary file 1 [file Supplementaryfile1.pdf]

SUPPLEMENTAL FIGURES

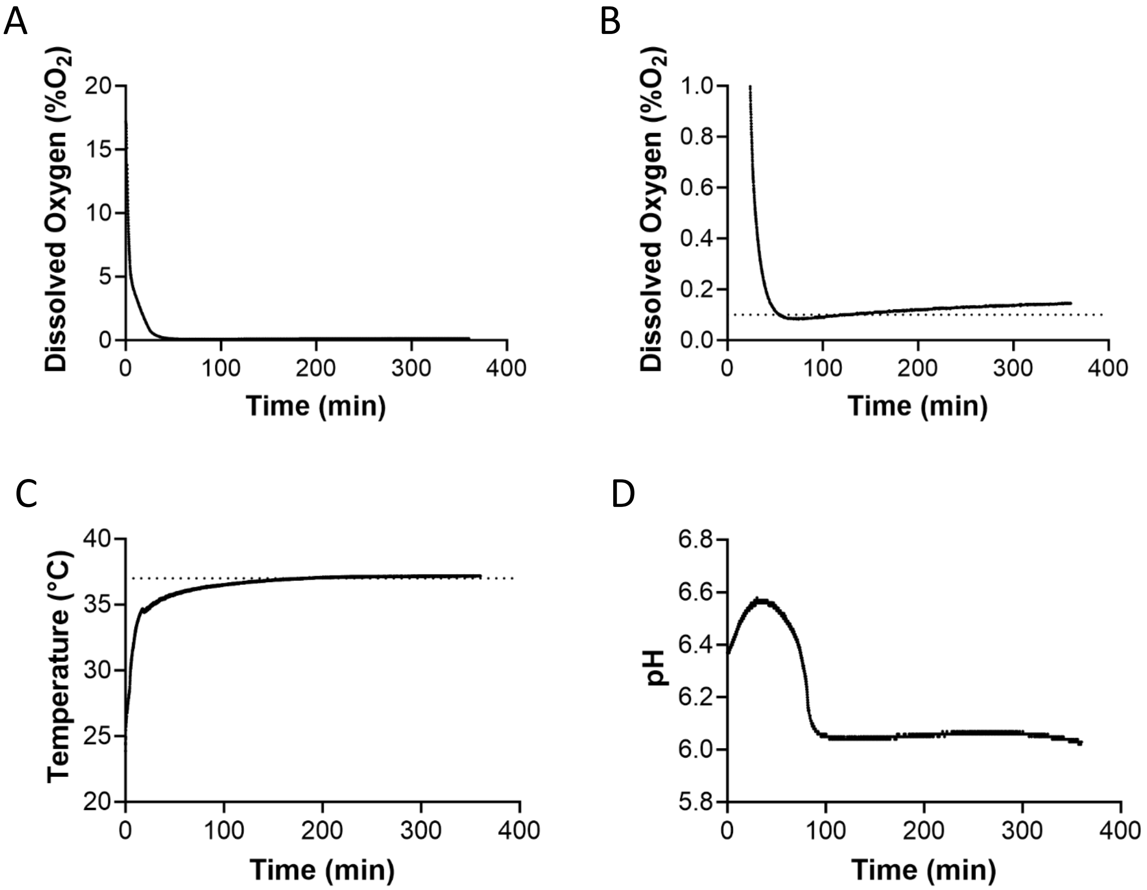

Supplemental Figure 1. Characterization of the ischemic microenvironment *in vitro*. (A) Measurement of percentage of dissolved oxygen in ischemic media within the bioreactor over time after being placed in a hypoxic chamber (O2 < 0.1%). **(B)** Graph of dissolved oxygen percentage on a magnified scale. **(C)** Measurement of chamber temperature over time. **(D)** Measurement of ischemic media pH over time.

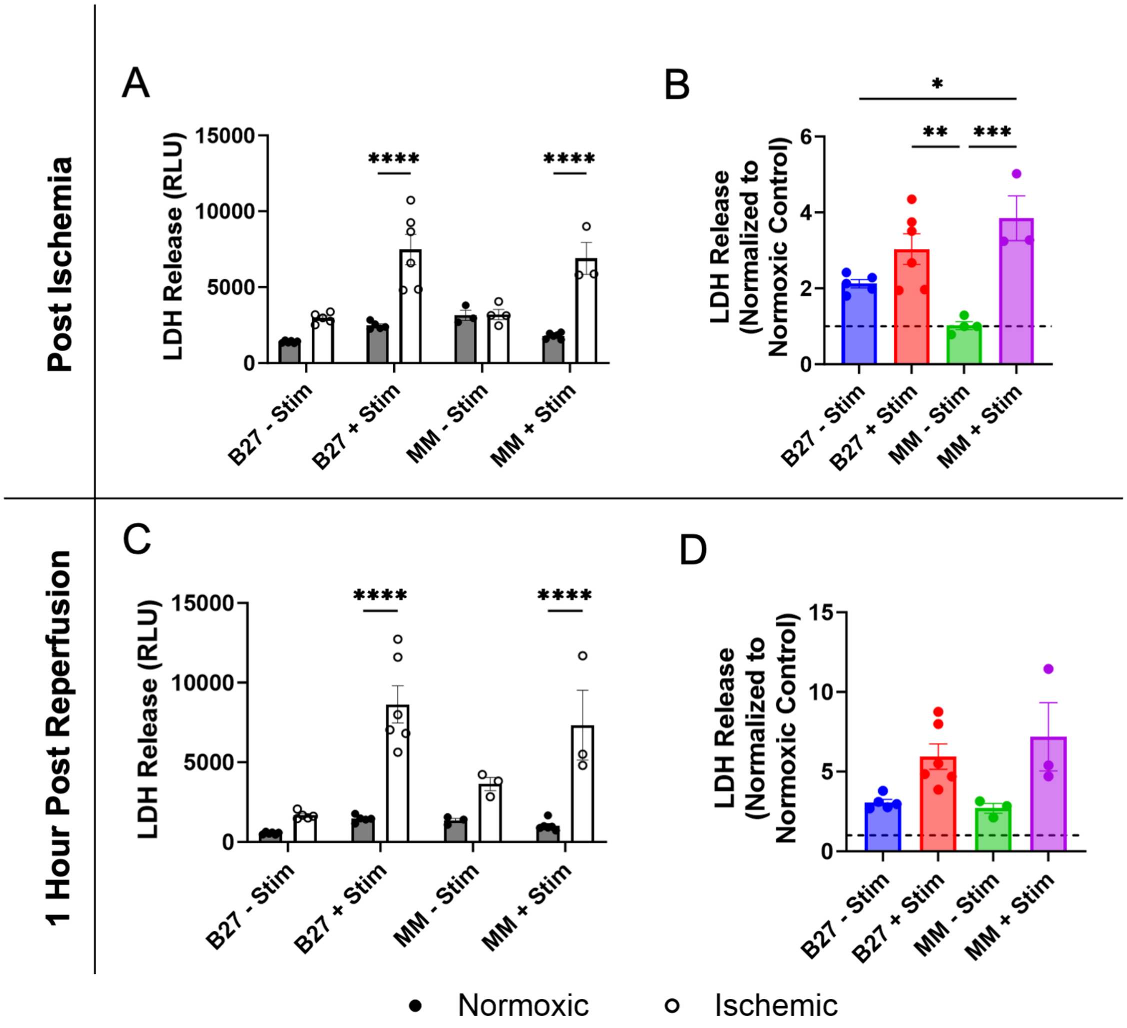

Supplemental Figure 2. Tissue conditioning influences cellular damage extent from ischemic injury at early timepoints. Cellular damage was assessed by lactate dehydrogenase (LDH) release of tissues subjected to ischemia compared to their normoxic controls at the end of the 6-hour ischemic period in (**A**) Direct comparison between all groups. (**B**) Direct comparison of ischemic groups when normalized to their normoxic controls to compare relative magnitude of damage. Assessment was repeated to assess damage from reperfusion injury one hour following reperfusion in (**C**) and (**D**) respectively. Data shown as mean ± SEM. * $p<0.05$, ** $p<0.01$, *** $p<0.001$, **** $p<0.0001$ (n = 3-6 tissues per condition).

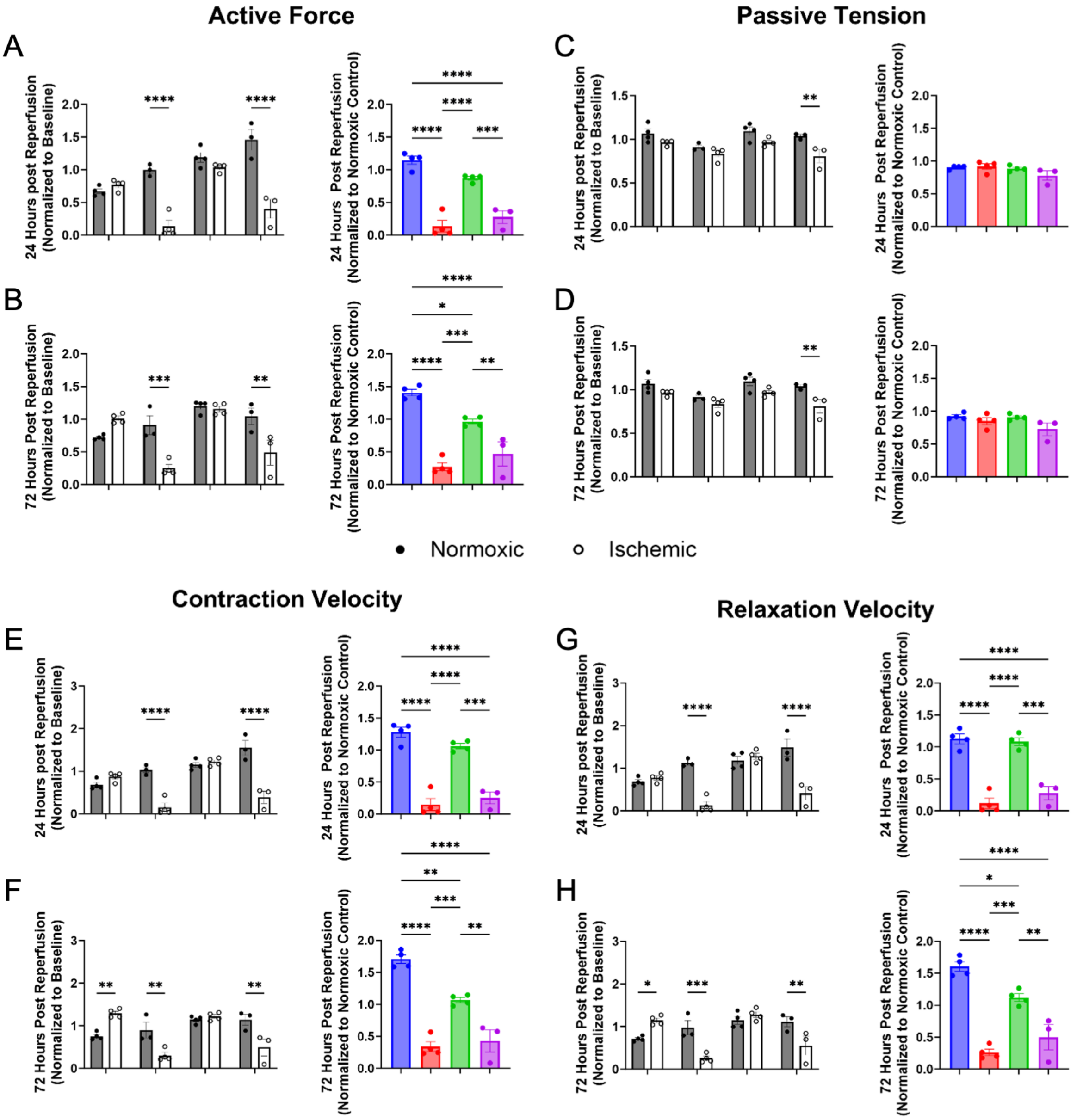

Supplemental Figure 3. Tissue conditioning impacts I/R effect on force production and contraction velocity. Active force of all tissue conditioning groups (B27-stim, B27+stim, MM-stim, and MM+stim) comparing ischemic tissues to their respective normoxic controls, as well as a direct comparison of the magnitude of change is quantified respectively at (A) 24 hours post reperfusion, (B) 72 hours post reperfusion. Relaxation velocity is similarly measured at (C) 24 hours post reperfusion, (D) 72 hours post reperfusion. Contraction velocity is quantified shown at (E) 24 hours post reperfusion and (F) 2 hours post reperfusion. Passive tension is similarly measured at (G) 24 hours post reperfusion, (H) 72 hours post reperfusion. Data shown as mean ± SEM. * $p<0.05$, ** $p<0.01$, *** $p<0.001$, **** $p<0.0001$.

Supplemental Table 1. Ischemic Media Composition

Component	Concentration (mM)	Product Information (Sigma catalog #)
NaCl	119	746398
KCl	12	P3911
NaH_2PO_4	1.2	RDD007
$MgSO_4$	1.3	M2643
$MgCl_2$ Hexahydrate	0.5	M2393
$CaCl_2$ Dihydrate	0.9	C5670
$NaC_3H_5O_3$ (Sodium Lactate)	20	71718
HEPES	5	H3375
